# Towards Environment Friendly Hydrothermally Synthesized Li^+^, Rb^+^, In^3+^ Intercalated Phosphotungstate (PW_12_O_40_) Thin Films

**DOI:** 10.3390/ma16030888

**Published:** 2023-01-17

**Authors:** Sameer N. Nadaf, Satish S. Patil, Vilasrao A. Kalantre, Sawanta S. Mali, Jyoti V. Patil, Chang Kook Hong, Sharadchandra S. Patil, Popatrao N. Bhosale, Sambhaji R. Mane

**Affiliations:** 1 Materials Research Laboratory, Department of Chemistry, Shivaji University, Kolhapur 416004, MS, India; 2 Department of Chemistry, Balasaheb Desai College, Patan 415206, MS, India; 3 Polymer Energy Materials Laboratory, School of Chemical Engineering, Chonnam National University, Gwangju 61186, Republic of Korea; 4 Optoelectronic Convergence Research Center, School of Chemical Engineering, Chonnam National University, Gwangju 61186, Republic of Korea; 5 Department of Physics, Shivaji University, Kolhapur 416004, MS, India

**Keywords:** polyoxometalates, hydrothermal, phosphotungstate (PW_12_O_40_), thin films, power conversion efficiency (PCE)

## Abstract

In the present investigation, a one-step hydrothermal approach is proposed to synthesize Li^+^, Rb^+^, and In^3+^intercalated PW_12_O_40_ (PTA) thin films. The photoelectrochemical performance of the deposited Li_3_PW_12_O_40_ (Li−PTA), Rb_3_PW_12_O_40_ (Rb−PTA), and In_3_PW_12_O_40_ (In−PTA) photocathodes were investigated using a two-electrode cell configuration of FTO/Li_3_PW_12_O_40_/(0.1 M I^−^/I^3−^)_aq._/Graphite. The energy band gaps of 2.24, 2.11, and 2.13 eV were observed for the Li−PTA, Rb−PTA, and In−PTA films, respectively, as a function of Li^+^, Rb^+^, and In^3+^. The evolution of the spinal cubic crystal structure with increased crystallite size was observed for Rb^+^ intercalation within the PTA Keggin structure, which was confirmed by X-ray diffraction (XRD). Scanning electron microscopy (SEM) revealed a modification in the surface morphology from a rod-like structure to a densely packed, uniform, and interconnected microsphere to small and large-sized microspheres for Li−PTA, Rb−PTA, and In−PTA, respectively. Compositional studies confirmed that the composing elements of Li, Rb, In, P, W, and O ions are well in accordance with their arrangement for Li^+^, Rb^+^, In^3+^, P^5+^, W^6+^, and O^2−^ valence states. Furthermore, the *J-V* performance of the deposited photocathode shows power conversion efficiencies (PCE) of 1.25%, 3.03%, and 1.62%, as a function of the incorporation of Li^+^, Rb^+^, and In^3+^ ions. This work offers a one-step hydrothermal approach that is a prominent way to develop Li^+^, Rb^+^, and In^3+^ ions intercalated PTA, i.e., Li_3_PW_12_O_40,_ Rb_3_PW_12_O_40_, and In_3_PW_12_O_40_ photocathodes for competent solar energy harvesting.

## 1. Introduction

Due to the limitations of non-renewable energy sources and the consumption of fossil fuels, the development and commercial synthesis of renewable energy sources have piqued the interest of today’s scholars in recent years. Because the sun is an infinite source of light energy, researchers are concentrating their efforts on developing new photovoltaic solar cells [1,2,3]. Polyoxometalates are used in the field of energy, such as solar cells [4] and lithium-ion batteries [5,6]. The most studied Keggin types of structures are [XM_12_O_40_]^n−^ and [PMo_12_O_40_]^3−^ [7]. Because these compounds are highly stable in air and water and can conduct reversible and steady electron transfer processes, they are attractive candidates for use in the energy field [8,9]. Although polyoxometalates offer attractive electrochemical characteristics, there have been comparatively few studies of polyoxometalate carried out on photo-electrochemical solar cells. A photoconversion efficiency (η) of 033% was reported in favor of (PW_12_/TiO_2_)_3_ thin films [10], and PW_12_-0.75 showed the highest (η) of 0.13% [11]. Recently, a Tl^+^-doped phosphomolybdic acid (TlPMA) absorber produced a conversion efficiency of 0.42% [12].

Polyoxometalates (POMs), a well-known class of inorganic nanoclusters with a metal–oxygen framework, have an inherent ability to accept electrons readily [13]. POMs can undergo reversible multielectron redox reactions while retaining their intact structure, making them an efficient electron scavenger to enhance the photoelectrochemical response [14]. Polyoxometalates (POMs), a class of molecular metal-oxo cluster compounds based primarily on Mo, W, and V elements, have demonstrated superior physicochemical properties and a wide range of applications [15,16]. POMs, in particular, have an intrinsic electron acceptor property that is likely to capture photogenerated electrons from the semiconductor conduction band (CB) and thus promote semiconductor power conversion efficiency. Yoon et al. reported that phosphotungstic acid played a vital role in improving the energy conversion efficiency of a TiO_2_ anode [17]. Researchers also carried out a systematic study in the fields of conductivity [18], photocatalysts [19,20], semiconductors [21], and polyoxometalates as absorber materials in photovoltaic (PV) cells.

Recently, our group investigated the incorporation of monovalent and trivalent metal ions into POMs films to enhance photoelectrochemical performance. These modifications of POMs upon doping could promote its photoelectrochemical performance; this is a promising strategy for enhancing electron–hole pair recombination, as a result, improving the power conversion efficiency of the PTA photocathode [22,23].

The partial exchange of protons in heteropoly acid with large cations such as Cs^+^, Rb^+^, NH_4_^+^, and K^+^ transforms a water-soluble acid with a low surface area (<5 m^2^g^−1^) into a water-insoluble salt with a surface area greater than 100 m^2^g^−1^ [24,25]. The presence of the counter cation was discovered to affect the relative stability of heteropoly acids. The heteropolyacids (HPAs) can be altered by exchanging protons with various metal or alkali metal ions. HPA salts are formed by the partial and complete exchange of heteropolyacid protons. As a result, the polyoxometalate materials exhibit significant improvement and optimization in the growth pathway for refining their optoelectronic and photovoltaic properties through the doping strategy. Doping is a technique that involves introducing small amounts of impurities into the lattice of a material, causing changes in the morphological, optostructural, and electrical properties of the parent material [26].

Polyoxometalate Keggin’s structure remains intact even after its protons are exchanged with various metal ions. The extent to which protons are exchanged with metal ions has a significant effect on these POMs’ photovoltaic, catalytic, and sensing properties. POMs that have been partially exchanged have been reported to have higher acidity than POMs that have been completely exchanged [27]. This is due to the increased surface area, which contributes to the protons’ high mobility.

Several research articles on the synthesis of monovalent metal-ion-exchanged polyoxometalate using solid-state ion exchange [28], batch reactor vessels [29], and layer-by-layer methods [30] have been published, but all of these methods produce materials in powder form, which is not suitable for solar cell application in terms of uniformity. Therefore, we have directly implemented the modified one-pot hydrothermal route to synthesize a uniform and adherent thin film, which is a simple and cost-effective procedure.

In the present investigation, the hydrothermal process was directly implemented to construct a Li_3_PW_12_O_40_ (Li-PTA), Rb_3_PW_12_O_40_ (Rb-PTA), and In_3_PW_12_O_40_ (In-PTA) absorber on fluorine-doped tin oxide (FTO) substrates using Na_2_-EDTA as a complexing agent. The main aim is to investigate the role of dopant monovalent Li^+^, Rb^+^, and trivalent In^3+^ ions in a phosphotungstate anion using a simple one-step hydrothermal process, as well as the effect on optostructural, compositional, and morphological properties. Furthermore, photoelectrochemical properties of directly synthesized Li_3_PW_12_O_40_ (Li-PTA), Rb_3_PW_12_O_40_ (Rb-PTA), and In_3_PW_12_O_40_ (In-PTA) thin films were investigated.

## 2. Experimental Details

### 2.1. Chemicals and Material Details

Thin films of Lithium (Li^+^), Rubidium (Rb^+^), and Indium (In^3+^)-intercalated phosphotungstate anions were synthesized using phosphotungstic acid (H_3_PW_12_O_40_) (PTA) and lithium chloride, rubidium sulfate, and indium sulfate, all of which were purchased from Himedia laboratories and used as sources of (PW_12_O_40_)^3−^ and Li^+^, Rb^+^, and In^3+^ ions, respectively. To allow for the gradual release of cations from the solution, the complex disodium salt of ethylenediaminetetraacetic acid (Na_2_−EDTA, 99.9%, S.D. Fine Chem., Hong Kong, China) was used. An ammonia solution (NH_3_, 25%, Thomas Baker, Mumbai, India) was used to keep the pH of the reaction bath stable. Using an FTO conducting substrate (~8 Ω/sq, Seoul, Republic of Korea), the photoelectrochemical application of Li^+^, Rb^+^, and In^3+^-intercalated phosphotungstate anions was investigated. To perform electrochemical reactions, a 0.1 M iodide/polyiodide solution was used to investigate the photoelectrochemical solar cell performance of Li−PTA, Rb−PTA, and In−PTA photoelectrodes. All solutions were produced with double-distilled water (DDW).

### 2.2. Preparation of Solutions

#### 2.2.1. Phosphotungstic Acid (H_3_PW_12_O_40_)

A 0.02 M H_3_PW_12_O_40_ solution was prepared by dissolving 5.76 g of H_3_PW_12_O_40_ in double-distilled water (DDW) and diluted to 100 mL with DDW.

#### 2.2.2. Lithium Chloride (LiCl)

A 0.1 M LiCl solution was made by dissolving 0.423 g of LiCl in double-distilled water (DDW) and diluting it to 100 mL with DDW.

#### 2.2.3. Rubidium Sulfate (Rb_2_SO_4_)

The 0.01 M Rb_2_SO_4_ solution was made by dissolving 0.267 g of Rb_2_SO_4_ in double-distilled water and diluting it to 100 mL with DDW.

#### 2.2.4. Indium (III) Sulfate (In_2_(SO_4_)_3_)

To make a 0.1 M In_2_(SO_4_)_3_ solution, we dissolved 0.517 g of In_2_(SO_4_)_3_in double-distilled water and diluted it to 100 mL with DDW.

#### 2.2.5. Ethylene Diamine Tetra Acetic Acid (EDTA)

A 0.1 M EDTA solution was made by dissolving 2.92 g of EDTA in double-distilled water and diluting it to 100 mL with DDW.

#### 2.2.6. Iodide/Polyiodide Electrolyte

Potassium iodide weighing 1.66 g was dissolved in a small amount of distilled water to make a 0.1 M solution. Then 1–2 iodine crystals and a pinch of KCl powder and d were added with double-distilled water to reach 100 mL.

### 2.3. Synthesis of Li^+^, Rb^+^, In^3+^ Doped PTA Thin Films

The synthesis of Li^+^, Rb^+^, and In^3+^ intercalated PTA thin films was deposited onto the FTO substrate using a one-step hydrothermal route. In the process, the appropriate volume of the 0.02 M H_3_PW_12_O_40_ solution was taken separately, and pH was adjusted to 5.2, 6.8, and 6.5 ± 0.2 for Li^+^, Rb^+^, and In^3+^, respectively. Using an NH_3_ solution, the colored metal-complex reaction solution changed to colorless and was then stirred for 10 min. Subsequently, an appropriate volume of freshly prepared EDTA complexed with a 0.1 M LiCl, In_2_(SO_4_)_3_, and 0.01 M Rb_2_SO_4_ salt (Li−EDTA, Rb−EDTA, and In−EDTA) solution was prepared and separately added drop-wise with continuous stirring to the above solution. Finally, the entire reaction mixture was transferred to a 40 mL Teflon-lined steel autoclave, and the precleaned FTO substrate was placed vertically in Teflon with the conducting side facing the solution.

For the hydrothermal synthesis of Li−PTA, Rb−PTA, and In−PTA, the autoclave was stored in the furnace at 90, 85, and 120 ± 2 °C for 3 h. After the desired period, the stainless-steel autoclave was naturally cooled to room temperature and the deposited films were dried for one hour at 100 °C and used for further characterization. Table 1 displays the optimized deposition parameters for Li−PTA, Rb−PTA, and In−PTA thin films.

### 2.4. Characterization of Li−PTA, Rb−PTA, and In−PTA Thin-Film Photocathode

The thickness of the Li−PTA, Rb−PTA, and In−PTA thin films was measured using a surface profilometer (AMBIOS Model-XP-1, Ambios Technology, Milpitas, CA, USA). The optical absorption spectra of the films were measured using a UV-Vis. spectrophotometer (Shimadzu Model-UV-1800, Shimadzu Corporation Analytical & Measuring Instruments Division, Kyoto, Japan) in the 300–700 nm range. An X-ray diffractometer was used to examine the structural properties of Li−PTA, Rb−PTA, and In−PTA films (Bruker Model-140 AXS D8, Bruker Ltd., Mannheim, Germany). Morphology and compositional data of Li-PTA, Rb-PTA, and In-PTA films were investigated using field emission scanning electron microscopy (FE-SEM) (MIRA3 LMH, TESCAN, Brno, Czech Republic, EU) coupled with energy-dispersive spectroscopy (EDS) (JEOL-JSM 6360A, JEOL Ltd., Tokyo, Japan). The analysis of valence states of constituted ions present in the Li−PTA, Rb−PTA, and In−PTA films was performed with X-ray photoelectron spectroscopy (XPS, Thermo Scientific, Model: Multilab-2000, Waltham, MA, USA).

A simple two-electrode cell configuration system was used to investigate the photoelectrochemical cell performance of Li−PTA, Rb−PTA, and In−PTA photocathodes. In the photo-electrochemical cell (PEC) setup, deposited Li−PTA, Rb−PTA, and In−PTA thin films serve as a photocathode, while the graphite plates serve as counter electrodes in a 0.1 M Iodide/polyiodide redox electrolyte solution under dark and light illumination from a 500 W tungsten lamp (light intensity of 100 mW/cm^2^). The electrode cell configuration of FTO/Rb-PTA|(0.1 M Iodide/polyiodide)_aq._|Graphite was used to assess the PEC performance. The spacing between the photocathode and the counter electrode was sustained up to ~1 cm. The electrochemical measurements were performed on an Autolab electrochemical workstation using NOVA software (Model: Autolab, PGSTAT 100 potentiostat, Metrohm, Herisau, Switzerland).

## 3. Results and Discussion

### 3.1. Proposed Growth and Reaction Pathway for Li−PTA, Rb−PTA, and In−PTA Thin Films

The surface morphology and crystal quality of Li−PTA, Rb−PTA, and In−PTA thin films depend on the deposition process of the films onto the substrate. A simple and easy low-cost hydrothermal approach is employing Na_2_-EDTA as a complexing agent to deposit Li−PTA, Rb−PTA, and In−PTA films onto a conducting fluorine-doped tin oxide (FTO) substrate. In this, the different metal ions were complexed with Na_2_-EDTA. Herein, the controlled release of metal ions at an optimized temperature and pressure is the reason for the chelating agent in this reaction. The Na_2_-EDTA is a useful organic complexing agent that helps to deposit high-quality Li−PTA, Rb−PTA, and In−PTA thin films by allowing different metal ions such as Lithium (Li^+^), Rubidium (Rb^+^), and (Indium) In^3+^ to slowly escape during the deposition process.

We controlled the deposition parameters of Li−PTA, Rb−PTA, and In−PTA films such as the temperature, pH, and precursor concentration to allow slowly released Li^+^, Rb^+^, and In^3+^ metal ions to react with phosphotungstate (PW_12_O_40_)^3−^ ions. The optimized temperature and pressure in the hydrothermal reaction promote a rapid rate of reaction by supplying heat energy to the reactant. As a result, a white-colored precipitate forms rather than a film. To avoid precipitation, metal ions are complexed with Na_2_−EDTA and decrease the precipitate with the formation of Li_3_PW_12_O_40_, Rb_3_PW_12_O_40_, and In_3_PW_12_O_40_ in powder form. At an optimized temperature and pressure, metal ions Li^+^, Rb^+^, and In^3+^ slowly dissociate from Li−EDTA, Rb−EDTA, and In−EDTA complexes inside the reaction and are deposited via ion-by-ion condensation followed by the Ostwald ripening process. In optimum conditions, smaller, thermodynamically unstable nuclei join one another to produce bigger stable crystals, according to the Ostwald ripening law [31]. When the ionic products of Li^+^, Rb^+^, and In^3+^ and (PMo_12_O_40_)^3−^ ions progress to the solubility product of the Li_3_PW_12_O_40_, Rb_3_PW_12_O_40_, and In_3_PW_12_O_40_ thin films, the deposition of Li−PTA, Rb−PTA, and In−PTA thin films on the conducting FTO substrate occur [32].

**Figure 1:**
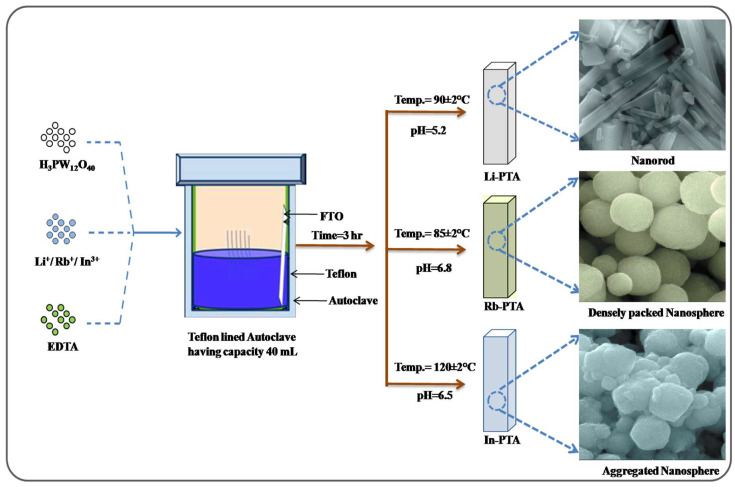
Graphical representation of possible growth mechanism of (i) Li_3_PW_12_O_40_, (ii) Rb_3_PW_12_O_40_, and (iii) In_3_PW_12_O_40_ thin film deposited by the single-step hydrothermal approach.

Hydrothermally synthesized Li_3_PW_12_O_40_, Rb_3_PW_12_O_40_, and In_3_PW_12_O_40_-based thin films exhibit good optoelectronic properties with Li^+^, Rb^+^, and In^3+^ intercalation, resulting in uniform, adherent, and improved film quality. The possible growth mechanism for the deposition of (i) Li−PTA, (ii) Rb−PTA, and (iii) In−PTA films by a cost-effective, simple, one-step hydrothermal technique is shown in Figure 1.

(i) The reaction pathway for the deposition of Li_3_PW_12_O_40_ films is as follows:

Initially, the formation of metal ion complex Li_2_−EDTA in an aqueous medium isdemonstrated in reaction (1)

2LiCl + Na_2_EDTA  →  [Li_2_−EDTA]^2−^ + 2NaCl (1)

At an optimal pH and reaction temperature, the H_3_PW_12_O_40_and Li_2_−EDTA complex dissociated and formed (PW_12_O_40_)^3−^and Li^+^, respectively, as shown in reactions (2) and (3) below.

H_3_PW_12_O_40_  →  (PW_12_O_40_)^3−^ (2)

Li_2_−EDTA]^2−^  →  2Li^+^ + (EDTA)^2−^ (3)

When ionic products of Li^+^ and (PW_12_O_40_)^3−^ ions exceeded and formed Li−PTA as a solubility product, the formation of a Li_3_PW_12_O_40_ film onto the FTO substrate was demonstrated in reaction (4).

(PW_12_O_40_)^3−^ + 3Li^+^  →  Li_3_PW_12_O_40_ (4)

(ii) The reaction pathway for the formation of Rb_3_PW_12_O_40_ films is as follows.

As demonstrated in reaction (5), the Rb_2_−EDTA complex is formed in an aqueous medium.

Rb_2_SO_4_ + Na_2_EDTA  →  [Rb_2_−EDTA]^2−^ + Na_2_SO_4_ (5)

At an optimized pH and reaction temperature, the H_3_PW_12_O_40_ and Rb_2_−EDTA complex dissociated and formed (PW_12_O_40_)^3−^ and Rb^+^, respectively, as shown in reactions (6) and (7) below.

H_3_PW_12_O_40_  →  (PW_12_O_40_)^3−^ (6)

[Rb_2_−EDTA]^2−^  →  2Rb^+^ + (EDTA)^2−^ (7)

When ionic products of Rb^+^ and (PW_12_O_40_)^3−^ ions exceeded and formed Rb−PTA as a solubility product, reaction (8) demonstrates the formation of the Rb_3_PW_12_O_40_ thin film on the FTO substrate.

 (PW_12_O_40_)^3−^ + 3Rb^+^  →  Rb_3_PW_12_O_40_ (8)

(iii) The reaction pathway for the formation of In_3_PW_12_O_40_ films is as follows.

The formation of an In_2_-EDTA complex in an aqueous medium is demonstrated in reaction (9).

In_2_(SO_4_)_3_ + Na_2_EDTA  →  [In_2_-EDTA]^2−^ + Na_2_(SO_4_)_3_ (9)

At an optimized pH and reaction temperature, the H_3_PW_12_O_40_ and In_2_−EDTA complex dissociated and formed (PW_12_O_40_)^3−^ and In^3+^, respectively, as shown in reactions (10) and (11) below.

H_3_PW_12_O_40_  →  (PW_12_O_40_)^3−^ (10)

[In_2_−EDTA]^2−^  →  2In^3+^ + (EDTA)^2−^ (11)

When ionic products of In^3+^ and (PW_12_O_40_)^3−^ ions exceeded and formed In-PTA as a solubility product, this resulted in the formation of In_3_PW_12_O_40_ thin film on the FTO substrate as indicated in reaction (12).

(PW_12_O_40_)^3−^ + 3In^3+^  →  In_3_PW_12_O_40_ (12)

### 3.2. Thickness Studies

A surface profilometer was used to calculate the thickness of the as-deposited Li−PTA, Rb−PTA, and In−PTA thin films, which were found to be 510, 620, and 570 nm, respectively. The thickness of the deposited films is controlled by uniform growth and surface morphology.

### 3.3. Optical Absorption Studies

**Figure 2:**
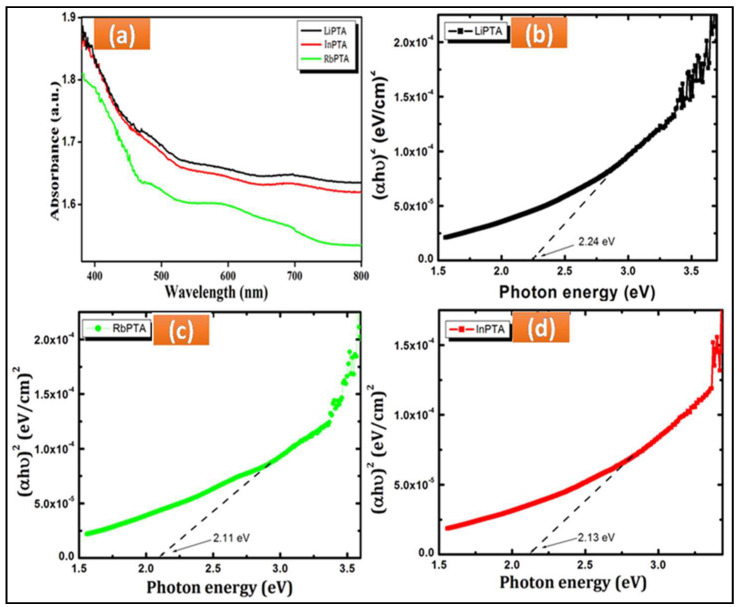
(**a**) Optical absorption spectra for Li−PTA, Rb−PTA, and In−PTA thin films and optical band gap of (**b**) Li−PTA (**c**) Rb−PTA, and (**d**) In−PTA thin films.

The film thickness of Li−PTA, Rb−PTA, and In−PTA films plays a crucial role in optical absorption studies, which are found to be 510, 620, and 570 nm, respectively. The synthesized Li−PTA, Rb−PTA, and In−PTA thin films indicate maximal light absorption within the range of 500–600 nm, which is observed in Figure 2a; as seen from the absorption spectra, slight shifting was observed in the absorption edge from Li^+^ to In^3+^ to Rb^+^ intercalation in the PTA film. The energy band gap of the Li−PTA, Rb−PTA, and In−PTA thin films decreases with an increase in thickness. Large-sized cations intercalate in PW_12_O_40_, increasing the grain-size expansion of Li−PTA, Rb−PTA, and In−PTA, which help to shrink the voids, leading to Li−PTA, Rb−PTA, and In−PTA absorbers. Furthermore, the decrease in band-gap energy of PW_12_O_40_ thin films might be due to additional energy levels introduced by Li^+^, Rb^+^, and In^3+^ ions, which are close to the valence band edge in the Eg of the host PW_12_O_40_, or a decrease in the energy associated with the direct and allowed transition, which causes the position of the absorption edge to shift to higher wavelength [33,34,35,36]. The band gap of deposited Li−PTA, Rb−PTA, and In−PTA films was determined by plotting the graph of (*αhυ*)^2^ versus (*hυ*), as shown in Figure 2b–d, which depends on Tauc Equation (13) [37].

(13) $$\alpha =\frac{A{\left(hv-Eg\right)}^{n}}{hv}$$

In which, ‘*h*’ is Planck’s constant, ‘*Eg*’ is the thin-film energy bandgap, exponent ‘*n*’ is the type of electronic transition, and ‘*A*’ is the parameter that depends on the probability of the electronic transition.

The optical absorption spectra of thin films have been studied to determine the absorption coefficient and optical energy gap. The optical properties of thin films are affected by their thickness and the chemical elements or compounds that comprise them. Photovoltaic, optoelectronics, electrochromic performance, solar cells, and photo-electrochemistry are some applications based on the optical absorption of thin films. Absorption is proportional to both the thickness of the film samples and the concentration of the absorbing material [26].

The calculated band gaps for Li−PTA, Rb−PTA, and In−PTA films were 2.24, 2.11, and 2.13 eV, respectively, as a result of Li^+^, In^3+^, and Rb^+^ ions intercalated in the PTA, which is lower than the parent PTA (3.24 eV). The energy band gap decreased owing to the increased thickness of Li−PTA, Rb−PTA, and In−PTA films. Moreover, a reduced band gap was observed in the PTA after Rb^+^ intercalation compared to Li^+^ and In^3+^, which is attributed to the increased thickness of synthesized films and the improved grain size. As a result, a densely packed and well-grown Rb_3_PW_12_O_40_ nanosphere film was formed. Rb−PTA has a narrower band gap than Li−PTA and In−PTA, which increases light absorption in photoelectrochemical cells [38].

### 3.4. Structural Analysis of Li−PTA, Rb−PTA, and In−PTA by XRD

**Figure 3:**
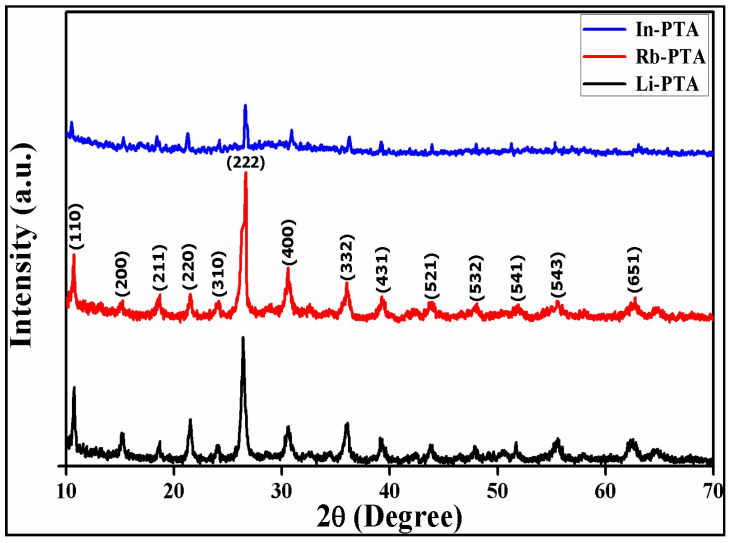
XRD patterns of Li−PTA, Rb−PTA, and In−PTA thin films.

The crystal structures of synthesized Li^+^, Rb^+^, and In^3+^-intercalated PTA films were assessed by the XRD patterns. The XRD analysis of synthesized Li−PTA, Rb−PTA, and In−PTA films were shown in Figure 3. As seen from Figure 3, the Li−PTA, Rb−PTA, and In−PTA films all show similar X-ray diffractograms of the body-centered cubic secondary Keggin structure with major diffraction peaks at 10.50–10.80, 15.15–15.32, 18.45–18.70, 21.27–21.53, 26.41–26.69, 30.59–30.85, 36.00–36.27, and 39.20–39.35° [39]. Compared to pure PTA, a slight shift in the prominent plane (222) towards higher 2θ values was observed in Li−PTA, Rb−PTA, and In−PTA, indicating the successful intercalation of Li^+^, Rb^+^, and In^3+^ in PTA. It is presumed that the Li^+^, Rb^+^, and In^3+^-intercalated PTA show similar symmetry but contracted unit cells.

The lattice parameters for the spinel cubic structure (a=b=c) for Li^+^, Rb^+^, and In^3+^-intercalated PTA were found to be 12.02, 11.52, and 11.90 Å, respectively [29,40]. This shrinkage of the unit cell can be described by the exchangeable protons present in the secondary Keggin structure in the form of dihydronium ions H_5_O_2_^+^ for hydrated Li^+^, Rb^+^, and In^3+^ cations, which are primarily responsible for the contraction in the unit cell parameter. This type of behavior was observed by Borghese et al. [41]. As a result, XRD demonstrates the successful intercalation of Li^+^, Rb^+^, and In^3+^ ions into PTA while preserving the Keggin structure. We calculated the crystallite size for Li−PTA, Rb−PTA, and In−PTA samples using Scherrer’s Equation (14) [42].

(14) $$D=\frac{0.94\;\lambda }{\beta \;\mathrm{COS}\;\theta }$$

where ‘D’ is the crystallite size (nm), ‘λ’ is the radiation source (CuKα = 1.5406 Å), ‘β’ is the full width at half maximum (FWHM) intensity (taken in radians), and ‘θ’ is Bragg’s diffraction angle (degree).

As shown in Table 2, the calculated crystallite sizes for Li−PTA, Rb−PTA, and In−PTA were 48.85, 72.03, and 52.54 nm, respectively. The larger rubidium cation size can be attributed to the increased crystallite size observed on Rb^+^ intercalated within the PTA Keggin structure. When metal ions intercalate, the reaction accelerates, resulting in the creation of a large number of nuclei and, as a result, a larger crystallite size, which is beneficial for lowering electron–hole pair recombination [26,31]. Crystallite-size-dependent structural parameters such as the dislocation density (δ) and microstrain (ε) were assessed using Equations (15) and (16) [43].

(15) $$\delta =\frac{1}{{\;D}^{2\;}}$$

(16) $$\varepsilon =\frac{\beta \;\mathrm{COS}\;\theta }{4}$$

It is clearly shown that crystallite size was increased in the Rb−PTA thin film compared to Li−PTA and In−PTA thin films due to the improved crystal quality and decreased recombination rate between the electrons and holes, resulting in increased *J_sc_* and *V_oc_* values and subsequently increased photoelectrochemical performance. Dislocation density (δ) and microstrain (ε) were calculated using Equations (15) and (16) and presented in Table 2. The estimated XRD parameters were correlated with the reported data. Furthermore, XRD analysis revealed that the Rb−PTA thin film has improved crystallinity when compared to Li−PTA and In−PTA thin films, lowering the grain boundary resistance and defect levels in Rb−PTA thin film. A reduced grain boundary in an Rb−PTA photocathode was beneficial for increasing power conversion efficiency.

### 3.5. Morphological Studies

**Figure 4:**
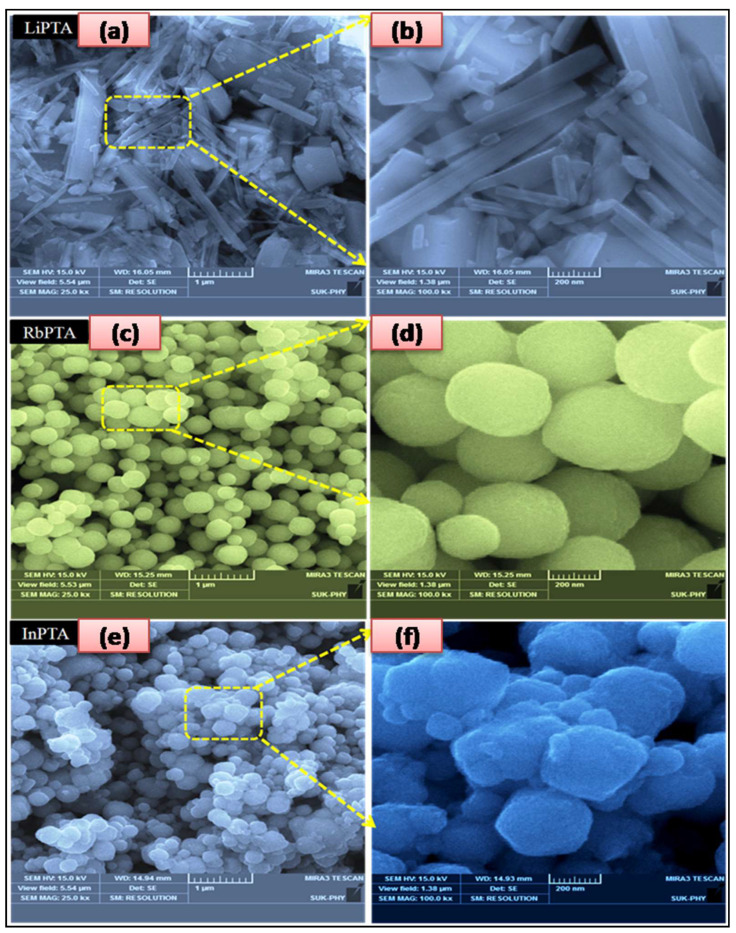
Low- and high-magnification SEM photographs of (**a**,**b**) Li−PTA, (**c**,**d**) Rb−PTA. and (**e**,**f**) In−PTA thin films.

The influence of Li^+^, Rb^+^, and In^3+^ ions on the surface morphology of PTA thin films was investigated using scanning electron microscopy (SEM). Figure 4a–f indicates SEM images of Li−PTA, Rb−PTA, and In−PTA samples at low and high magnifications. Figure 4a,b show low- and high-magnification SEM photographs of the Li−PTA film with irregular-sized rod-like structures and an average grain size of ~0.08–0.32 μm. Low- and high-magnification SEM pictures of the Rb−PTA thin film confirm the formation of a densely packed, well-grown, uniform-sized and -shaped, interconnected, microspherical surface morphology with an average grain size of ~0.50–0.70 μm, as shown in Figure 4c,d. Figure 4e,f show low- and high-magnification SEM pictures of the In−PTA thin film and reveal the creation of small and large-sized micro-spherical surface morphology with an average grain size of ~0.35–0.60 μm, respectively.

Hydrothermally produced microsphere and rod-shaped surface morphologies are significant for improving optical, electrical, and electronic properties, as well as increasing the surface area for light absorption in solar cells. Overall, the morphological analysis shows that developing the Rb−PTA surface morphology with interconnected microspheres of increasing grain size, which reduces grain boundary resistance, is useful for the further enhancement of electrochemical properties [39].

### 3.6. Compositional Studies

**Figure 5:**
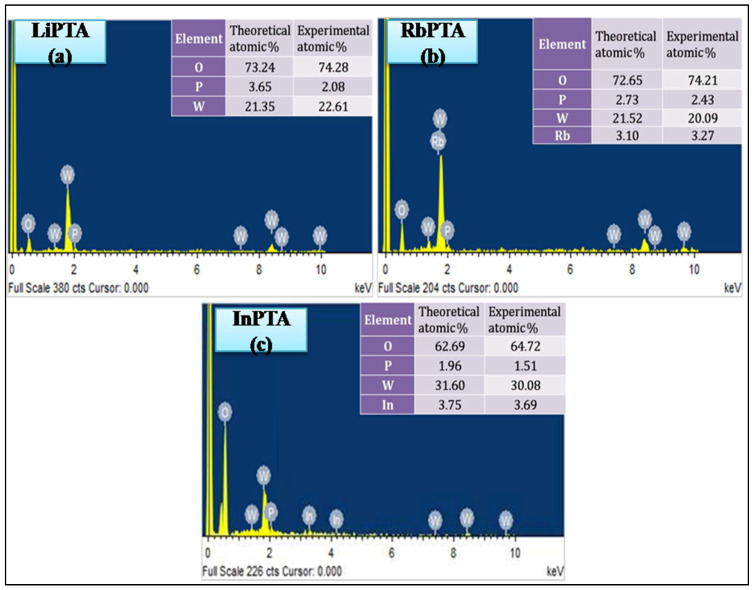
Energy-dispersive X-ray spectra of (**a**) Li−PTA, (**b**) Rb−PTA, and (**c**) In−PTA thin films.

The chemical composition of the Li^+^, Rb^+^, and In^3+^ ion-doped phosphotungstate anion influences the output of PEC performance because it reveals the stoichiometry of the produced Li−PTA, Rb−PTA, and In−PTA photocathodes, hence EDAX analysis is performed for Li−PTA, Rb−PTA, and In−PTA. Figure 5a–c depict the elemental composition of constituent elements present in Li−PTA, Rb−PTA, and In−PTA thin films, respectively. The EDAX spectra of the deposited Li−PTA thin film are shown in Figure 5a, and the peaks that arise at binding energies (B.E.) of 0.5, 2.1, and 1.4 KeV confirm the presence of oxygen, phosphorus, and tungsten elements, respectively. Lithium has the atomic number of (Z = 3) and it has very low energy and is not detected in EDAX analysis due to the detection limit. The EDAX spectra of the deposited Rb−PTA thin film are shown in Figure 5b. Peaks confirm the existence of oxygen, phosphorus, tungsten, and rubidium elements at 0.5, 2.1, 1.4, and 1.8 KeV binding energies, respectively. Figure 5c depicts the EDAX spectra of the deposited In-PTA thin film. The peaks appear at binding energies of 0.5, 2.1, 1.4, and 3.3 KeV, which validate the occurrence of oxygen, phosphorus, tungsten, and indium, respectively. The EDAX analysis indicates that theoretical and experimental atomic percentages of Li−PTA, Rb−PTA, and In−PTA samples are consistent, tabulated in the right inset of Figure 5a–c [44]. Distinct atomic percentages of O, P, and W were observed in deposition; this is due to the reactivity of monovalent and trivalent ions to bare PW_12_O_40_.

**Figure 6:**
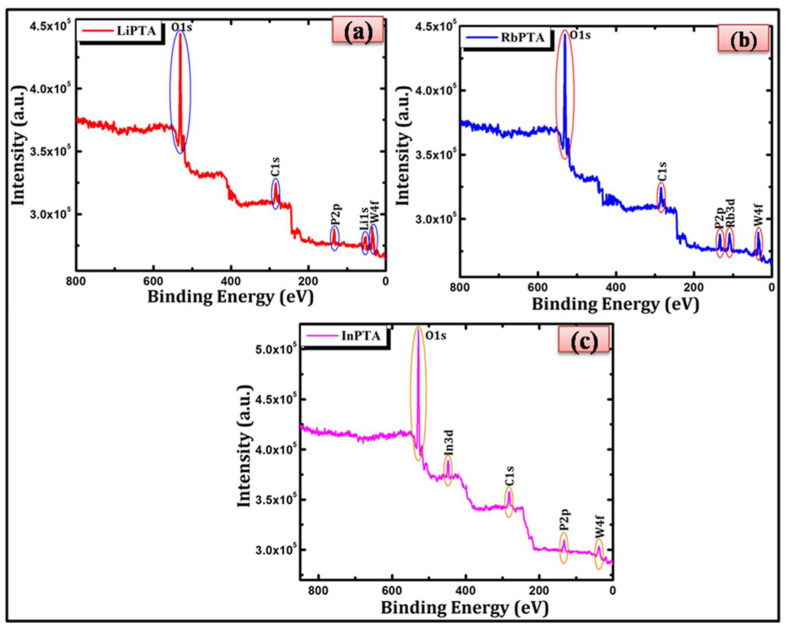
XPS survey spectra of (**a**) Li−PTA, (**b**) Rb−PTA, and (**c**) In−PTA thin films.

Figure 6 shows the XPS analyses of Li−PTA, Rb−PTA, and In−PTA films that were analyzed to investigate the chemical parameters of the Li_3_PW_12_O_40_, Rb_3_PW_12_O_40_, and In_3_PW_12_O_40_ thin films. Figure 6a demonstrates the XPS survey spectrum of the Li−PTA sample, which shows the occurrence of Li, P, W, and O elements with their corresponding binding energy (B.E.) positions, which confirms the presence of lithium (1+), phosphorus (5+), tungsten (6+), and oxygen (2−) oxidation states. Figure 6b represents the XPS survey spectrum of the Rb−PTA sample, which shows the existence of Rb, P, W, and O elements with their respective binding energies positions, which confirms the presence of rubidium (1+), phosphorus (5+), tungsten (6+), and oxygen (2−) oxidation states. Figure 6c depicts the XPS survey spectrum of the In−PTA sample, which affirms the presence of In, P, W, and O elements with their corresponding(B.E.) positions, which confirms the presence of indium (3+), phosphorus (5+), tungsten (6+), and oxygen (2−) oxidation states. In addition, the survey spectra of Li−PTA, Rb−PTA, and In−PTA contain a carbon peak, which is assigned as adventitious carbon.

Overall, the survey spectra for Li−PTA, Rb−PTA, and In−PTA -based thin films show they are composed of elements Li, Rb, In, P, W, and O ions are reliable in their arrangement for Li^+^, Rb^+^, In^3+^, P^5+^, W^6+^, and O^2−^ valence states, confirming that synthesized Li−PTA, Rb−PTA, and In−PTA thin films are consistent with their stoichiometric formula, Li_3_PW_12_O_40_, Rb_3_PW_12_O_40_, and In_3_PW_12_O_40_, respectively.

### 3.7. Photoelectrochemical Performance of Li−PTA, Rb−PTA, and In−PTA Thin Films

**Figure 7:**
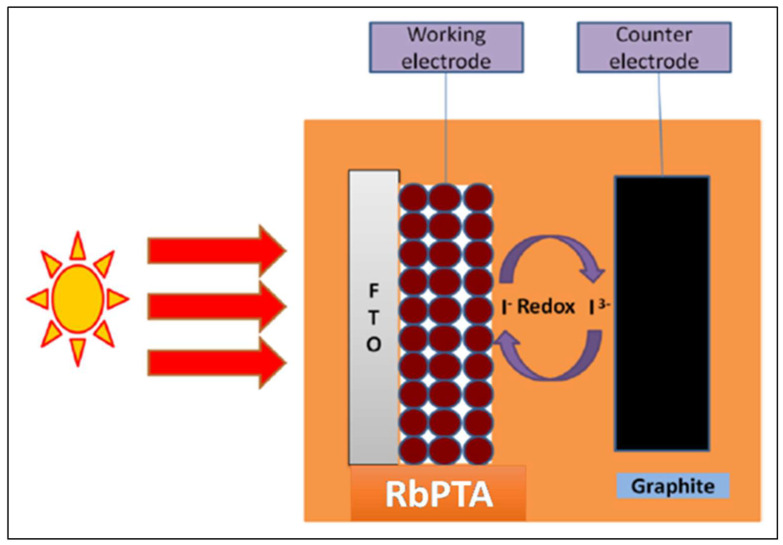
Schematic representation of Rb−PTA photocathode.

The photoelectrochemical performance of Li−PTA, Rb−PTA, and In−PTA photocathodes was measured with a simple, cost-efficient, liquid junction photoelectrochemical (PEC) cell. Figure 7 depicts a schematic illustration of the Rb−PTA photocathode in the PEC cell configuration. The photoelectrodes in the PEC cells were made up of Li−PTA, Rb−PTA, and In−PTA thin films. A graphite blade acted as a counter electrode, and an electrolyte solution of 0.1 M Iodide/polyiodide was utilized for the electrochemical process. The photoelectrochemical cell is represented as:FTO/Rb−PTA |Iodide/polyiodide (0.1 M)|Graphite.

**Figure 8:**
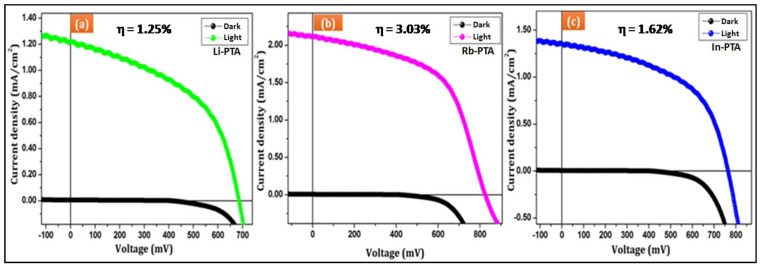
Current density–voltage (*J-V*) characteristics of deposited (**a**) Li−PTA, (**b**) Rb−PTA, and (**c**) In−PTA.

The *J-V* characteristics of the Li−PTA, Rb−PTA, and In−PTA photocathodes were analyzed on the auto-lab electrochemical workstation shown in Figure 8a–c. When the photocathode was exposed to light, it absorbed incident light. As a result, an electronic transition appeared from the valence band (VB) to the conduction band (CB). Current density–voltage measurements were performed on deposited Li−PTA, Rb−PTA, and In−PTA samples without and with illumination from a 100 mW/cm^2^ filament lamp. Figure 8 shows that, under dark conditions, Li−PTA, Rb−PTA, and In−PTA photoelectrodes behave similarly to rectifier diodes, and under light irradiation, it is observed that the magnitude of the open-circuit voltage increases with positive polarity, confirming that Li−PTA, Rb−PTA, and In−PTA photoelectrodes are *p*-type semiconductors.

From the *J-V* curves, the fill factor (*FF*) and power conversion efficiency (*η*%) of Li^+^, Rb^+^, and In^3+^-intercalated PTA films were evaluated using Equations (17) and (18) [45].

(17) $$FF=\frac{J_{max}\times V_{max}}{J_{sc}\times V_{oc}}$$

(18) $$\eta \%=\frac{J_{sc}\times V_{oc}}{P_{in}}\times FF\times 100$$

where ‘*J_max_*’ is the maximum output current, ‘*V_max_*’ is the maximum output voltage, ‘*J_sc_’* is the short-circuit current, ‘*V_oc_’* is the open circuit voltage, and ‘*P_in_*’ is the input light intensity of a tungsten lamp (100 mW/cm^2^).

As seen from the *J-V* analysis, there was an increment in current density upon Li^+^, Rb^+^, and In^3+^-intercalated PTA films. The calculated power conversion efficiency for Li−PTA, Rb−PTA, and In−PTA was 1.25%, 3.03%, and 1.62%, respectively. An improvement in PEC was seen due to the intercalation of Li^+^, Rb^+^, and In^3+^ in PTA. Table 3 summarizes all of the photo-electrochemical cell performance data, which shows lower Voc values observed in the *J-V* measurements. This might be due to recombination processes in the photoelectrochemical cell [46,47].

As seen from Figure 8, when Li^+^, Rb^+^, and In^3+^ were intercalated in PTA, an increase in current density and open-circuit voltage was seen as a result of improving the PEC performance. The increase in PEC output is due to the formation of compactly and densely packed uniform films of Li−PTA, Rb−PTA, and In−PTA, respectively. Rb−PTA presents highly uniform and compact film formation that reduces the number of grain boundaries and provides enough space to absorb photons compared to Li−PTA and In−PTA thin films. Furthermore, the uniform film surface shows close exposure between the electrolyte the and electrode along with a sufficient surface area for photogenerated electrons, while the less uniform film is present in Li−PTA and In−PTA, which licks the photocurrent, thus forming a low current that accomplishes the lower conversion efficiency as compared to Rb−PTA. Overall, the Rb−PTA photocathode exhibits a better photoelectrochemical performance, attributed to improvements in the densely packed film and increased thickness, providing enough surface for solar energy absorption and diminishing the rate of electron–hole pair recombination, which is useful for photoabsorption in photoelectrochemical solar cells.

## 4. Conclusions

To fabricate the Li−PTA, Rb−PTA, and In−PTA thin films onto FTO substrates, a one-step, cost-effective, non-toxic hydrothermal route was used. Optical and structural studies revealed that Rb−PTA thin films exhibit direct and appropriate types of transitions with lower energy bandgap, i.e., 2.11 eV, when compared to Li−PTA and In−PTA thin films. The surface morphology of Li^+^, Rb^+^, and In^3+^ intercalation in PTA differs, resulting in irregular rods, interconnected microspheres, and agglomerated microspheres, respectively. The presence of Li, Rb, In, P, W, and O ions with valence states of Li^+^, Rb^+^, In^3+^, P^5+^, W^6+^, and O^2−^ is confirmed by compositional data. The PEC study confirms the generation of conversion efficiencies of 1.25%, 3.03%, and 1.62% for Li−PTA, Rb−PTA, and In−PTA photocathodes, respectively. Improvement in the power conversion efficiency (PCE) for Rb−PTA was observed, attributed to the optimal bandgap produced after Rb^+^ ion intercalation. Moreover, from the achieved results, the one-step hydrothermal approach is a better alternative than other thin-film deposition techniques.

## Figures and Tables

**Table 1:** Optimized parameters for the synthesis of Li−PTA, Rb−PTA, and In−PTA thin films.

Sample Code	Bath Composition	pH	Temperature (°C)	Deposition Time (h)
Li−PTA	20 mL of 0.02 M H_3_PW_12_O_40_^+^ liquor NH_3_^+^ 10 mL of Li-EDTA complex were added to the above solution and the whole reaction mixture was put into a Teflon-lined stainless-steel autoclave with a capacity of 40 mL.	5.2 ± 0.2	90 ± 2 °C	3 h
Rb−PTA	20 mL of 0.02 M H_3_PW_12_O_40_^+^ liquor NH_3_^+^ 10 mL of Rb-EDTA complex were added to the above solution and the whole reaction mixture was put into a Teflon-lined stainless-steel autoclave with a capacity of 40 mL.	6.8 ± 0.2	85 ± 2 °C	3 h
In−PTA	20 mL of 0.02 M H_3_PW_12_O_40_^+^ liquor NH_3_^+^ 10 mL of In-EDTA complex were added to the above solution and the whole reaction mixture was put into a Teflon-lined stainless-steel autoclave with a capacity of 40 mL.	6.5 ± 0.2	120 ± 2 °C	3 h

**Table 2:** Structural parameters of Li−PTA, Rb−PTA, and In−PTA thin films.

Sample Code	Crystallite Size (nm)	Dislocation Density (δ) (Line m^−2^) × 10^14^	Microstrain (ε) (line^−2^ m^−4^) × 10^−4^	Experimental d-Spacing Value (Å)
Li−PTA	48.85	3.71	6.68	3.36
Rb−PTA	72.03	1.92	4.81	3.33
In−PTA	52.54	3.27	5.96	3.34

**Table 3:** Photoelectrochemical parameters of Li−PTA, Rb−PTA, and In−PTA photocathode.

Sample Code	Eg (eV)	*J_sc_* (mA cm^−2^)	*V_oc_* (mV)	*J_max_* (mA cm^−2^)	*V_max_* (mV)	*FF*	*η*%
Li−PTA	2.24	1.23	690	0.92	418	0.44	1.254
Rb−PTA	2.11	2.20	827	1.67	571	0.50	3.032
In−PTA	2.13	1.35	770	1.05	465	0.46	1.627

## Data Availability

Not applicable.
